# Novel reporter mouse models useful for evaluating *in vivo* gene editing and for optimization of methods of delivering genome editing tools

**DOI:** 10.1016/j.omtn.2021.03.003

**Published:** 2021-03-05

**Authors:** Hiromi Miura, Jurai Imafuku, Aki Kurosaki, Masahiro Sato, Yongjie Ma, Guisheng Zhang, Akiko Mizutani, Kenya Kamimura, Channabasavaiah B. Gurumurthy, Dexi Liu, Masato Ohtsuka

**Affiliations:** 1 Department of Molecular Life Science, Division of Basic Medical Science and Molecular Medicine, Tokai University School of Medicine, Kanagawa, Japan; 2 Center for Matrix Biology and Medicine, Graduate School of Medicine, Tokai University, Kanagawa, Japan; 3 Section of Gene Expression Regulation, Frontier Science Research Center, Kagoshima University, Kagoshima, Japan; 4 Department of Pharmaceutical and Biomedical Sciences, College of Pharmacy, University of Georgia, Athens, GA, USA; 5 Faculty of Health and Medical Science, Teikyo Heisei University, Tokyo, Japan; 6 Division of Gastroenterology and Hepatology, Graduate School of Medical and Dental Sciences, Niigata University, Niigata, Japan; 7 Department of General Medicine, Niigata University School of Medicine, Niigata, JAPAN; 8 Mouse Genome Engineering Core Facility, University of Nebraska Medical Center, Omaha, NE, USA; 9 Department of Pharmacology and Experimental Neuroscience, College of Medicine, University of Nebraska Medical Center, Omaha, NE, USA

**Keywords:** CRISPR, gene therapy, *in vivo* gene editing, EGFP, transgenic mice, genome editing efficiency, hydrodynamic gene delivery

## Abstract

The clustered regularly interspersed palindromic repeats (CRISPR) system is a powerful genome-editing tool to modify genomes, virtually in any species. The CRISPR tool has now been utilized in many areas of medical research, including gene therapy. Although several proof-of-concept studies show the feasibility of *in vivo* gene therapy applications for correcting disease-causing mutations, and new and improved tools are constantly being developed, there are not many choices of suitable reporter models to evaluate genome editor tools and their delivery methods. Here, we developed and validated reporter mouse models containing a single copy of disrupted *EGFP* (ΔEGFP) via frameshift mutations. We tested several delivery methods for validation of the reporters, and we demonstrated their utility to assess both non-homologous end-joining (NHEJ) and via homology-directed repair (HDR) processes in embryos and in somatic tissues. With the use of the reporters, we also show that hydrodynamic delivery of ribonucleoprotein (RNP) with *Streptococcus pyogenes* (Sp)Cas9 protein mixed with synthetic guide RNA (gRNA) elicits better genome-editing efficiencies than the plasmid vector-based system in mouse liver. The reporters can also be used for assessing HDR efficiencies of the *Acidaminococcus sp.* (As)Cas12a nuclease. The results suggest that the ΔEGFP mouse models serve as valuable tools for evaluation of *in vivo* genome editing.

## Graphical abstract

**Figure:**
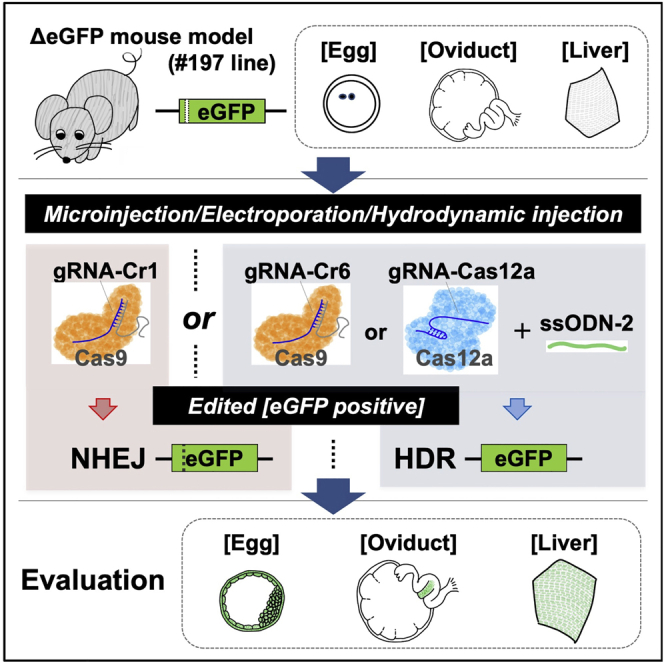

## Teaser

CRISPR-Cas is a revolutionary genome-editing technology that is making big impacts in biomedical research and gene therapy. Miura and colleagues developed reporter mice that can emit green fluorescence only in the edited cells or tissues and showed their utility as the much-needed *in vivo* tools for evaluating CRISPR-Cas technology.

## Introduction

Programmable nucleases, such as zinc finger nucleases (ZFNs), transcription activator-like effector nucleases (TALENs), and clustered regularly interspaced short palindromic repeats/CRISPR-associated protein 9 (CRISPR/Cas9), have emerged as well-established tools for targeted manipulation of the genomic sequence. They are used for insertion and deletion of nucleotides (indels) by non-homologous end joining (NHEJ) and for targeted genome replacement via homology-directed repair (HDR).^1^ Among these, the CRISPR-Cas9 system has been widely used, due to its simplicity and flexibility.^2^ The CRISPR-Cas9 system consists of a Cas9 endonuclease and a short RNA sequence called guide RNA (gRNA), which directs the Cas9 endonuclease to a specific genomic site.^2^

Some studies have investigated the applications of CRISPR-Cas9 to gene therapy.^3^, ^4^, ^5^ The successful gene therapy treatments by *in vivo* genome editing used animal models bearing Duchenne muscular dystrophy, hereditary tyrosinemia, or hemophilia B disease.^6^, ^7^, ^8^, ^9^, ^10^ Even though a lot of progress was made in developing the CRISPR tool, there are still challenges to make the tool more efficient.^11^ For example, many attempts have been made to enhance HDR efficiency for precise gene correction by insertion of a repair DNA template,^12^, ^13^, ^14^ since the HDR pathway is generally less efficient compared to the NHEJ pathway.^15^^,^^16^ Reporter animal models can be very useful for evaluating CRISPR, or its newer and improved versions, for gene therapy applications. Such reporter models will be useful for identification of factors affecting genome editing efficiency in clinically relevant conditions and for assessing the efficiency of delivery of genomic editing machinery *in vivo*.

In this study, we developed mouse models containing a frameshift mutation of the EGFP reporter (ΔEGFP), disabling its fluorescence capability. The ΔEGFP mouse lines were tested for genome editing in embryonic cells by microinjection and in somatic cells by either electroporation or hydrodynamic delivery, and genome editing efficiencies of different CRISPR-Cas9 platforms (plasmid and ribonucleoprotein [RNP]) were compared in liver cells. Green florescence detected in cells of the ΔEGFP mice indicate successful EGFP editing, which can be quantified. Our results demonstrate that ΔEGFP mice serve as valuable tools for *in vivo* genome editing research.

## Results

### Generation of a ΔEGFP transgenic (Tg) mouse

**Figure 1:**
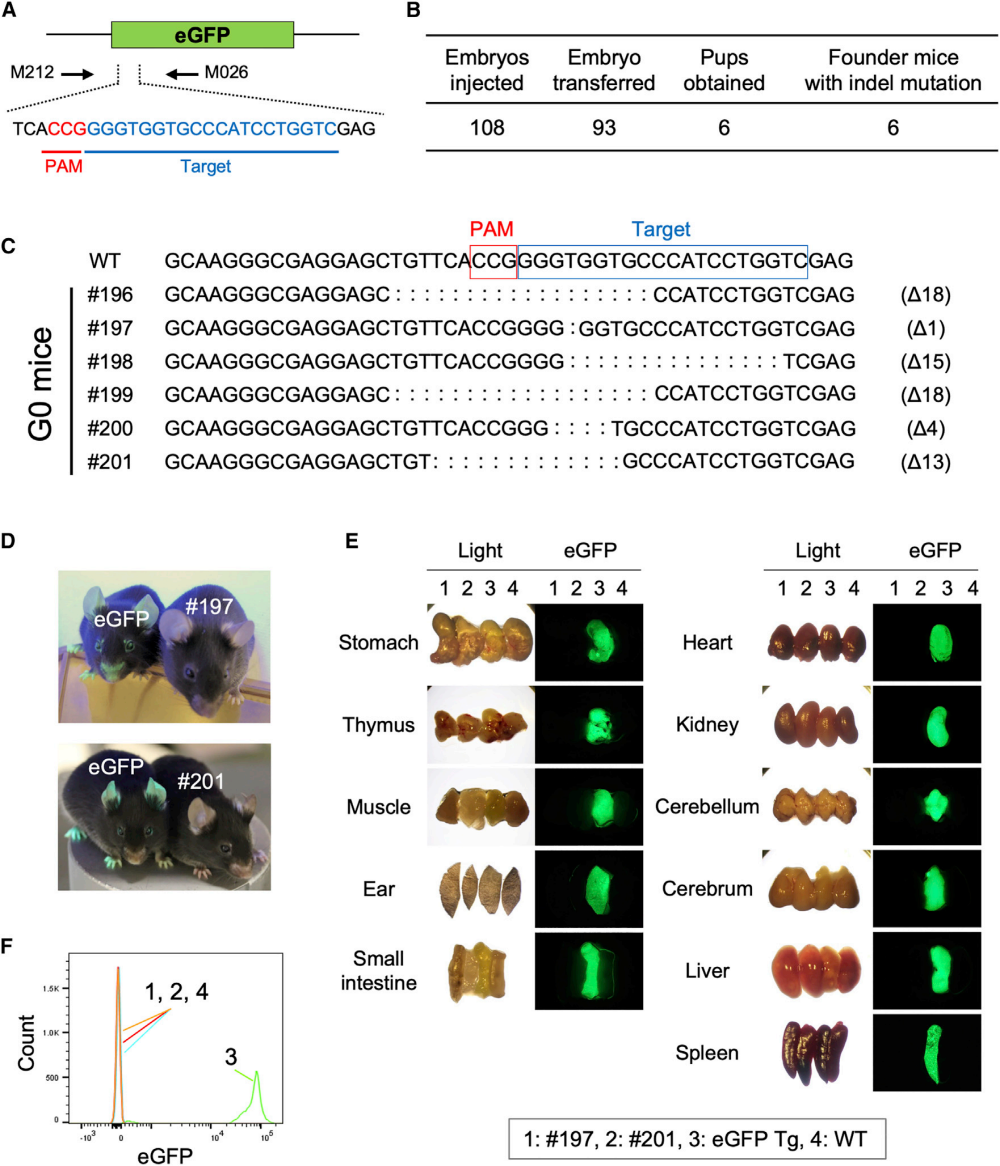
Creation of ΔEGFP transgenic (Tg) mice (A) Schematic of the targeting strategy to create frameshift mutations in the *EGFP* sequence in the Tg line. The primer set used for genotyping is shown by arrows (M212 and M026; Table S5). The sequence recognized by gRNA and location of PAM are shown in blue and red, respectively. (B) Microinjection data of creating ΔEGFP Tg mice. (C) Mutated *EGFP* alleles found in the resultant G0 mice. The sizes of deletion are indicated as Δnt on the right-side of the sequences. WT, wild-type. (D) EGFP fluorescence in ΔEGFP mouse lines (#197 and #201), together with the original EGFP Tg line (EGFP) after photographing under a hand-held UV lamp (365 nm). Each mouse has a single copy configuration of “CAG-EGFP (or ΔEGFP)-poly(A)” cassette at the *Rosa26* locus. (E) EGFP fluorescence in organs/tissues of mouse lines (#197, #201, EGFP Tg, and WT). (F) EGFP fluorescence intensity in the FACS-sorted spleen cells isolated from mouse lines (#197, #201, EGFP Tg, and WT).

To generate fluorescence-based reporter mouse models suitable for *in vivo* testing, we first disrupted the *EGFP* coding sequence in our previously developed single-copy CAG-*EGFP*-pA Tg mouse line (EGFP Tg) (Figure S1).^17^ Microinjection of a solution containing single gRNA (sgRNA) targeting *EGFP* (sgRNA-EGFP) (Table S1; Figure 1A) and *Cas9* mRNA (SpCas9, derived from *Streptococcus pyogenes*) into 108 zygotes derived from the EGFP Tg produced six live pups after zygote implantation. Sequencing the target region revealed that all pups (6/6, 100%) had deletion mutations in the *EGFP* sequence (Figure 1B), ranging from 1 to 18 nucleotides (nt) (Figure 1C). Among these, two founder mice (termed #197 and #201 ΔEGFP Tg [carrying 1 nt and 13 nt deletion, respectively]) were bred to establish lines for further experiments. We confirmed the loss of EGFP fluorescence in several organs (Figures 1D and 1E) and performed fluorescence-activated cell sorting (FACS) analysis of splenic cells to confirm loss of EGFP fluorescence at cellular levels, whereas the fluorescence in the parental line was readily detected (Figure 1F). These results confirmed that the ΔEGFP Tg mice lost EGFP fluorescence in all tissues.

### Restoration of EGFP fluorescence in the ΔEGFP Tg mice

**Figure 2:**
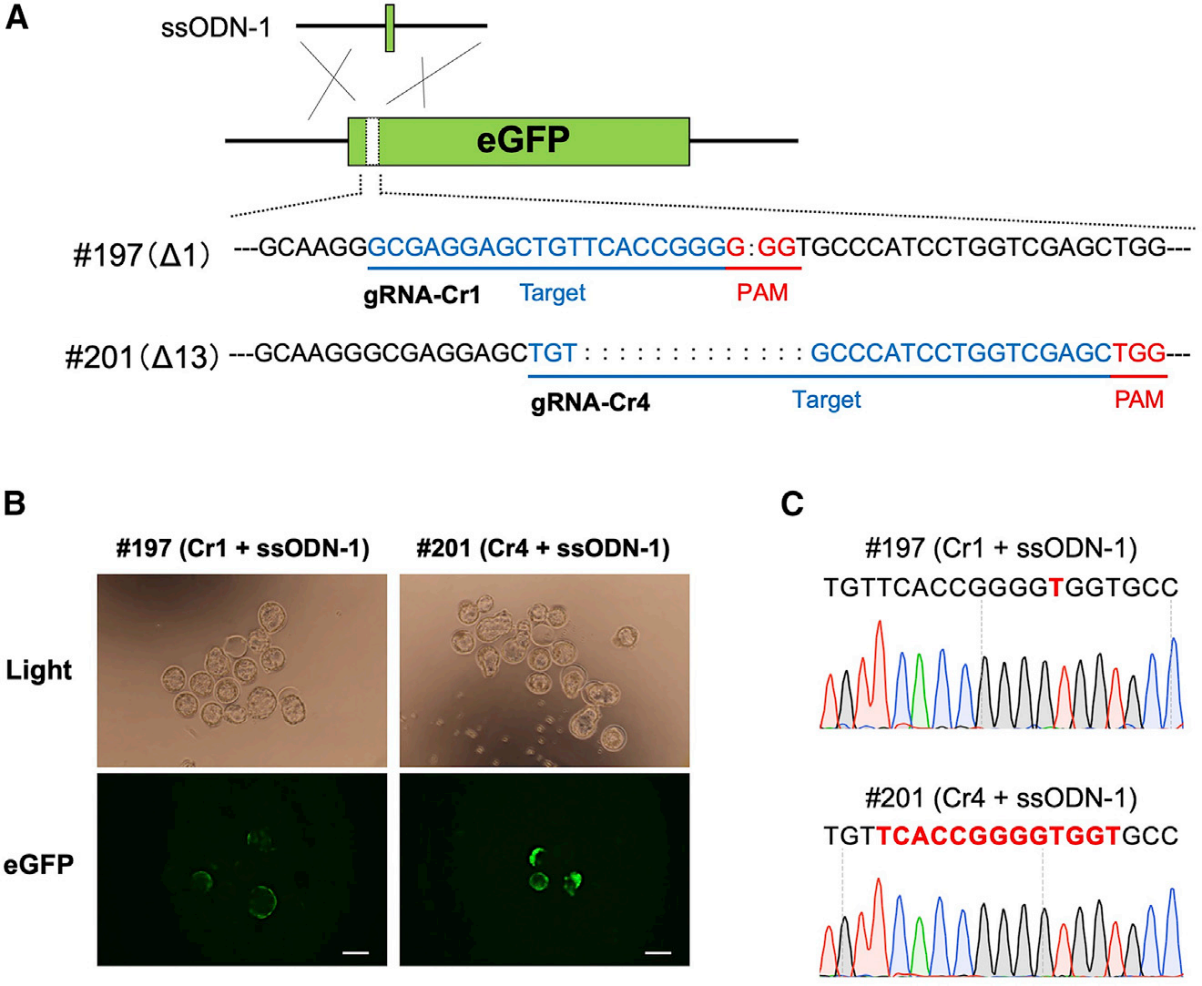
ssODN-based knock-in enables restoration of EGFP fluorescence in eggs derived from ΔEGFP mice (A) Schematic of the strategy showing gene correction in the ΔEGFP mice using ssODN-1. The gRNA target sequences are shown in blue, and PAMs are shown in red. The gRNA-Cr1 and gRNA-Cr4 were used for #197 and #201 mouse lines, respectively. The symbol “:” indicates 1 bp deletion in the EGFP parental sequence. (B) Detection of EGFP fluorescence in the blastocysts after zygote injection of CRISPR-Cas9 components. Scale bars, 100 μm. (C) Representative chromatograms obtained from direct sequencing of PCR products, each for a single blastocyst exhibiting EGFP fluorescence shown in (B). The corrected nucleotide sequences are shown in red letters.

We next tested whether the mutated alleles can be corrected in the zygotes of lines #197 and #201 by microinjection of Cas9 mRNA/sgRNAs/ssODN-1. Two sgRNAs were designed: gRNA-Cr1 was specific to the mutated *EGFP* sequence in #197 and gRNA-Cr4 to that in #201 (Figure 2A; Table S1). The injected zygotes were cultured *in vitro*, and the gene correction was assessed by EGFP fluorescence in the blastocysts. Three of the 11 blastocysts (27%) derived from line #197 and three of the 15 blastocysts (20%) derived from line #201 exhibited EGFP fluorescence (Figure 2B). Sequencing of the targeted regions in all of the blastocysts showed precise correction of the *EGFP* sequences, suggesting successful knock-in of the ssODN-1 donor template (Figure 2C).

**Figure 3:**
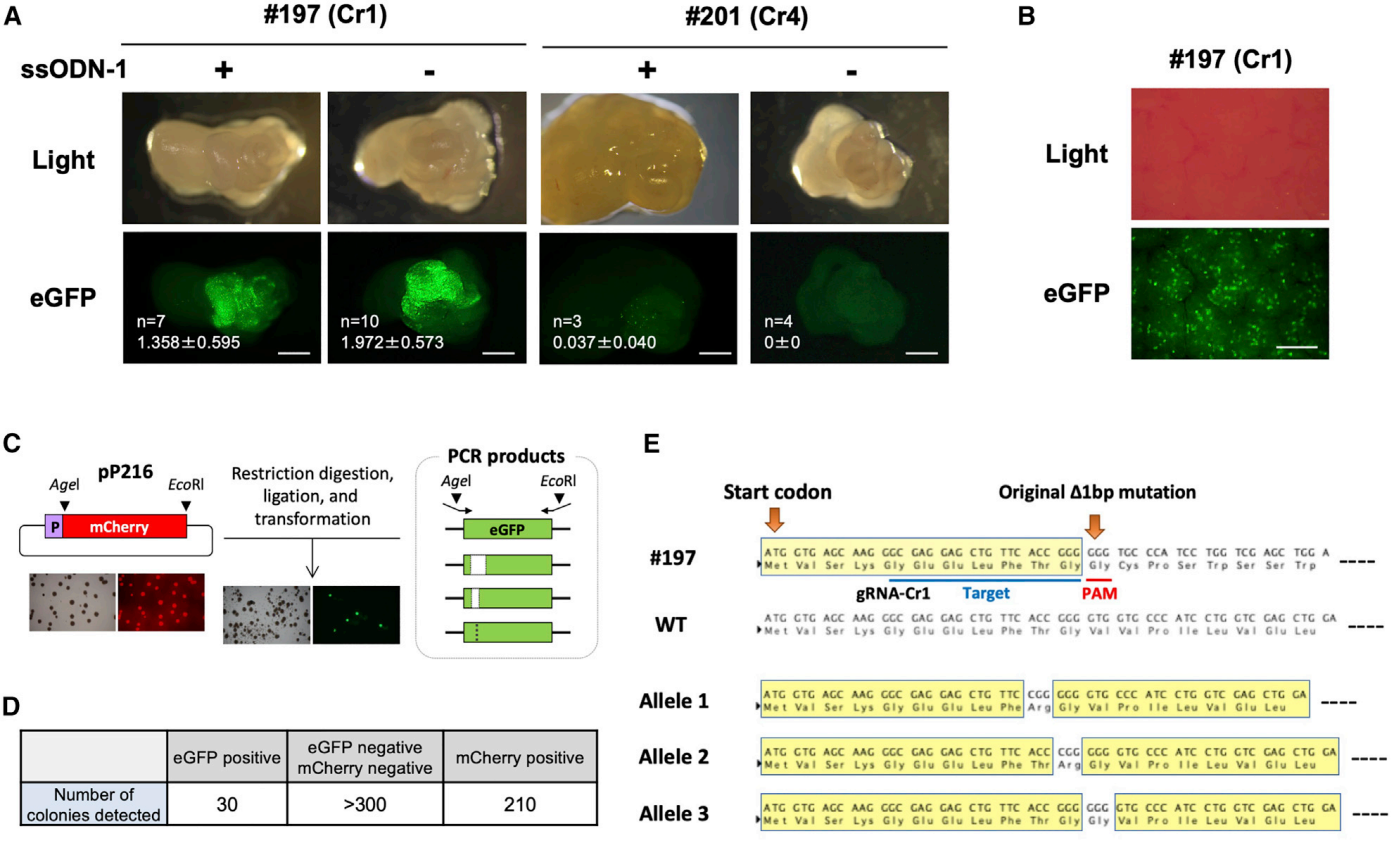
*In vivo* restoration of EGFP fluorescence (A) Detection of EGFP fluorescence in the oviduct after intra-oviductal injection of CRISPR-Cas9 components and subsequent *in vivo* electroporation. Transfection with RNP (Cas9 protein and gRNA-Cr1 or -Cr4) + ssODN-1 was designated as “+,” and that with RNP alone as “−.” The number of oviducts examined (n) and the ratio of green area (% area) in the image are shown in the lower left side of each image. Scale bars, 1 mm. (B) Detection of EGFP fluorescence in the liver of #197 mouse line, 3 days after hydrodynamics-based delivery of the plasmid pP189 (that expresses Cas9 and sgRNA-Cr1). Scale bar, 500 μm. (C) Schematic of cloning strategy for detection of edited *EGFP* sequences that are capable of showing fluorescence. Plasmid pP216 digested with AgeI and EcoRI was ligated with PCR-amplified *EGFP* fragments after digestion with the same enzymes. EGFP fluorescent-positive colonies were picked, and *EGFP* sequences were examined. (D) The number of colonies obtained in the cloning experiment (C). (E) The nucleotide sequence and deduced amino acids for the *EGFP* gene from #197 line, WT, and clones isolated from the fluorescent colonies. Yellow boxes show the regions that share the same amino acid sequence with that of the WT EGFP protein.

To examine whether restoration of EGFP fluorescence can also be achieved in somatic cells, we performed intra-oviductal injection of CRISPR-Cas9 components (Cas9 protein and trans-activating CRISPR RNA [tracrRNA]/gRNA-Cr1 or gRNA-Cr4, with or without ssODN-1) into ΔEGFP female mice (#197 or #201) and subsequently performed *in vivo* electroporation of the entire oviducts, a procedure previously developed for transfection of oviductal epithelia.^18^ The EGFP fluorescence was examined at either 3 days or 1 week after electroporation. Results presented in Figure 3A showed fluorescent cells in oviducts derived from both lines #197 and #201. Unexpectedly, electroporation in the absence of ssODN-1 also restored fluorescence in the line #197, which is suggestive of gene correction via the NHEJ repair pathway (Figures 3A and S2), whereas the oviducts from line #201 did not restore fluorescence. To test if the restoration of fluorescence occurs in the absence of ssODN-1, even when the Cas9 and gRNA were delivered in the form of plasmid DNA, we performed a hydrodynamics-based procedure to deliver pP189 plasmid vector containing Cas9 and sgRNA-Cr1 expression cassettes into #197 mice (Figure S1), and the animals were analyzed after 3 days. EGFP-positive cells were observed in the liver, suggesting that functional EGFP was restored through in-frame indel mutations created by the NHEJ repair mechanism (Figure 3B).

To confirm that fluorescence was indeed derived from indel mutations introduced within the mutated *EGFP* gene, we next examined the nucleotide sequences of *EGFP* in oviductal epithelial cells showing fluorescence. The target region was PCR amplified and sub-cloned into the pP216 vector (Figure 3C). After transformation of the sub-cloned vectors into *E. coli*, the resultant colonies were assessed for fluorescence. Green fluorescent colonies were observed (Figures 3C and 3D), and sequencing analysis of three fluorescent clones demonstrated that all clones contained *de novo* indel mutations that were in-frame with the sequence of *EGFP* (Figure 3E). Taken together, gRNA-Cr1/Cas9 administration into the somatic tissue of line #197 induces restoration of EGFP fluorescence through NHEJ without ssODN-1.

### Restoration of EGFP fluorescence using additional gRNAs

**Figure 4:**
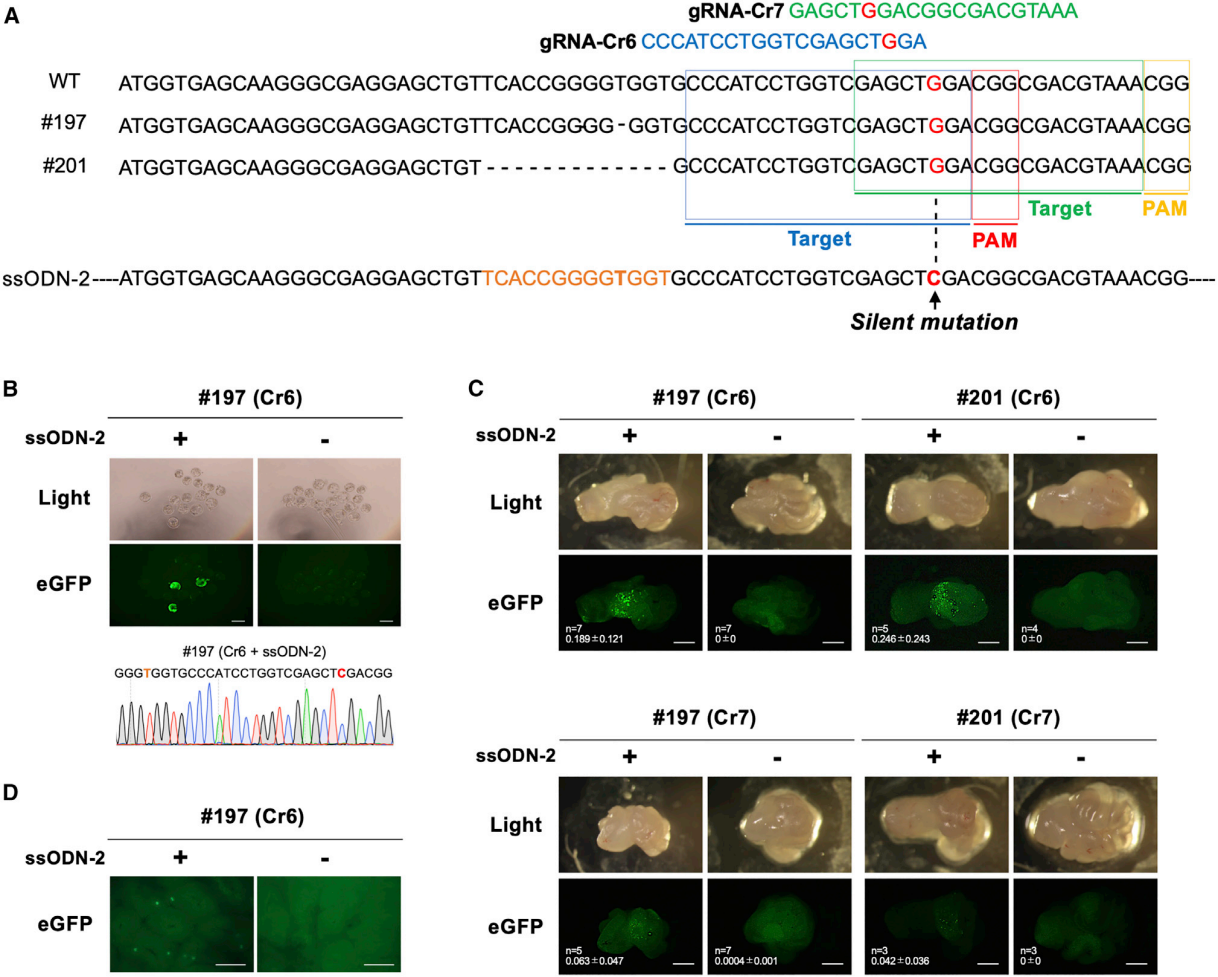
Designing and evaluation of gRNAs for HDR-mediated correction of the ΔEGFP mutation (A) Schematic of the strategy showing correction of the ΔEGFP mutation using ssODN-2. The gRNA target sequences for gRNA-Cr6 and gRNA-Cr7 are indicated by blue and green rectangles, and PAMs are by red and yellow rectangles, respectively. The ssODN-2 donor contains repair sequences suitable for both of the ΔEGFP lines #197 and #201 and a silent mutation (shown in red) that inhibits re-cutting by Cas9. (B) Detection of EGFP fluorescence in blastocysts after zygote injection of CRISPR-Cas9 components. Injection with RNP + ssODN-2 was designated as “+,” and that with RNP alone as “−.” Scale bars, 100 μm. Representative chromatogram obtained from direct sequencing of a PCR product. (C) Detection of EGFP fluorescence in the oviduct after intra-oviductal injection of CRISPR components and subsequent *in vivo* electroporation. Transfection with RNP (containing Cas9 protein and gRNA-Cr6 [upper panel] or gRNA-Cr7 [lower panel]) + ssODN-2 was designated as “+,” and that with RNP (containing Cas9 protein and gRNA-Cr6 or -Cr7) alone as “−.” Two lines (#197 [left panel] and #201 [right panel]) were used. The number of oviducts examined (n) and the ratio of green area (% area) in the image are shown in the lower left side of each image. Scale bars, 1 mm. (D) Detection of EGFP fluorescence in the liver of #197 line, 3 days after hydrodynamics-based administration of CRISPR components (pP217 [plasmid containing gRNA-Cr6 expression cassette] +/− ssODN-2). Transfection with pP217 + ssODN-2 was designated as “+,” and that with pP217 alone as “−.” Scale bars, 500 μm.

We next designed two more gRNAs, called gRNA-Cr6 and gRNA-Cr7, targeting the downstream regions of the mutated sequence of *EGFP* cDNA for both #197 and #201 lines (Figure 4A; Table S1). The gRNA-Cr6 and gRNA-Cr7 cleaved 21 and 33 bases away from the deletion region (of 1 nt) in the line #197, and they cleaved 18 and 30 bases away from the deletion region (of 13 nt) in the line #201, respectively. We designed another ssODN (ssODN-2), harboring silent mutation to prevent re-cleavage after correct insertion, as a repair template for the Cr-6 and Cr-7 gRNAs. We expected three outcomes using this set of gRNAs and ssODN donor: if the guide (Cr6 or Cr7) does not cleave, the EGFP will not be restored; if the guide cleaves and if HDR does not occur, the new indels created at the second site on *EGFP* will still not restore fluorescence; and if the guide cleaves, and ssODN-mediated HDR occurs, the fluorescence gets restored. We also expected that NHEJ-based restoration of fluorescence is unlikely to occur because indel mutation introduced into the first beta-strand region of the EGFP protein by these guides (Cr6 or Cr7) could disrupt EGFP function, even if it corrects the reading frame of the ΔEGFP region. Therefore, with this set of gRNAs (Cr6 or Cr7 and the ssODN-2), the restoration of fluorescence can only be detected if HDR-based editing occurs. To confirm that the gRNAs and ssODN-2 function as expected in the ΔEGFP Tg mice, we first microinjected gRNA-Cr6/Cas9 protein into zygotes derived from line #197, with and without ssODN-2. Fluorescent blastocysts were seen with efficiency of 20% (3/15) (Figure 4B). The genomic DNA isolated from the fluorescent blastocysts were subjected to PCR, followed by DNA sequencing. The results show expected correction of the *EGFP* sequence (Figure 4B).

We next examined whether the gRNAs Cr6 or Cr7 induce restoration of fluorescence by NHEJ- or HDR-based editing in an *in vivo* experiment. Intra-oviductal injection of gRNA (for Cr6 or Cr7) and Cas9 protein and subsequent *in vivo* electroporation did not restore fluorescence in oviductal cells in either #197 or #201 lines, whereas injection of the components together with ssODN-2 and subsequent electroporation led to a few fluorescent oviductal epithelial cells (Figures 4C and S2), suggesting that the restoration occurred only via HDR and not via NHEJ (i.e., restoration occurred only when the delivery components consisted of ssODN-2, and no fluorescent cells were seen without ssODN-2) (Figure 4C). The fluorescent spots of oviductal cells were slightly abundant in the experiments with gRNA-Cr6 compared to that with gRNA-Cr7 (Figure 4C), which could be either because Cr6 is 12 bases closer to the frameshift-deletion regions than the Cr7 or because of the differences in the cleavage efficiencies of these two guides. We also confirmed the presence of fluorescent cells in the liver when the gRNA-Cr6/Cas9 expression vector (pP217) was delivered together with ssODN-2 (Figure 4D). Taken together, these data suggest that gRNA (Cr6 and Cr7) and ssODN-2 can be used for evaluation of HDR-based knock-in efficiency.

### Comparison of genome editing efficiency in different formats of CRISPR reagents: RNP versus plasmid DNA

**Figure 5:**
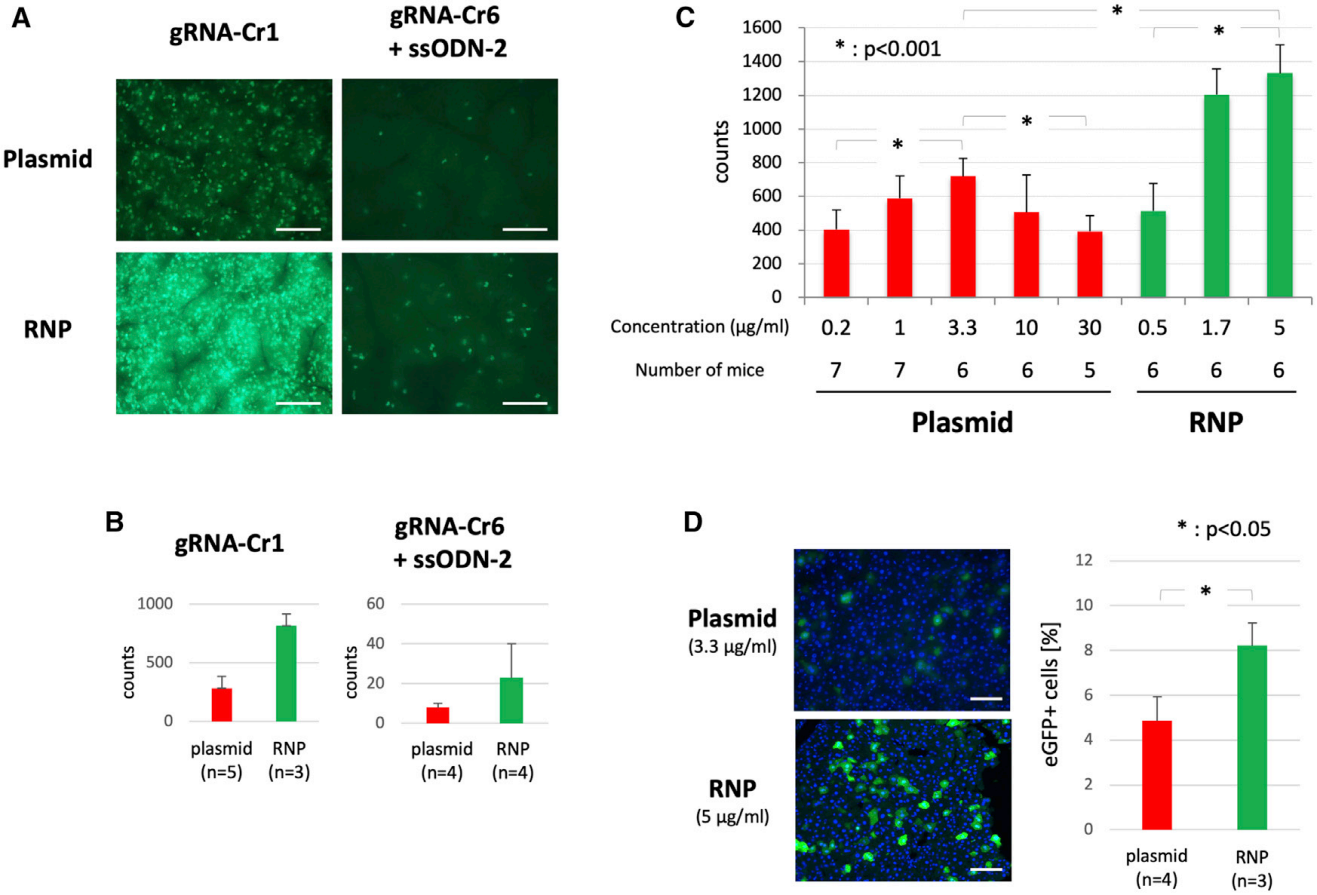
Comparison of *in vivo* genome editing efficiency using different CRISPR-Cas9 formats (A) Detection of EGFP fluorescence in the liver after hydrodynamic injection of CRISPR-Cas9 components (plasmid [10 μg/mL] or RNP containing Cas9 protein [5 μg/mL] and gRNA with or without ssODN-2). pP189 (for Cas9 and sgRNA-Cr1 expression) and pP217 (for Cas9 and sgRNA-Cr6 expression) were used as plasmid DNA. Images were acquired with system A (see Materials and methods). Scale bars, 500 μm. (B) The numbers of green spots in the images, such as shown in (A), were counted using ImageJ software. (C) Detection of EGFP fluorescence in the liver after hydrodynamic injection of various concentrations of CRISPR-Cas9 components (using gRNA-Cr1). Images for data analyses were acquired with system B (see Materials and methods), and the numbers of green spots were counted using ImageJ software. (D) Observation of the frozen liver sections 3 days after hydrodynamic injection of CRISPR-Cas9 components (plasmid [3.3 μg/mL] or RNP containing Cas9 protein [5 μg/mL] and gRNA-Cr1). The nuclei were stained with DAPI (blue). The ratio of EGFP-positive cells over DAPI fluorescence (counted using ImageJ software) was calculated. Scale bars, 100 μm.

We next compared the efficiency of two forms of CRISPR-Cas9 editing systems: the RNP and Cas9 plasmid vector in mouse liver using hydrodynamic delivery. 4 days after the hydrodynamic delivery of gRNA-Cr1/Cas9 (5 μg/mL protein for RNP and 10 μg/mL for plasmid) into the #197 mouse line, the fluorescence was observed in liver cells (with system A [see Materials and methods]). The fluorescence intensity seen in the liver was significantly higher with RNP compared to the plasmid vector carrying the Cas9 gene and gRNA-Cr1 expression cassettes in both NHEJ-based editing (by gRNA-Cr1) and HDR-based editing (by gRNA-Cr6) (Figure 5A). Semiquantitatively, the number of fluorescent spots via RNP delivery was approximately 2.9-fold higher than that achieved by plasmid DNA (282 ± 103 [plasmid DNA] and 818 ± 98 [RNP] for Cr1 and 8 ± 2 [plasmid DNA] and 23 ± 17 [RNP] for Cr6) (Figure 5B).

To determine a concentration that yields the highest editing outcome for each CRISPR format, hydrodynamic injections of various concentrations of CRISPR components (using gRNA-Cr1) were performed for both RNP and plasmid formats (0.5~5 μg/mL protein and 0.2~30 μg/mL plasmid, respectively). 3 days after the hydrodynamic delivery, the fluorescence was observed in liver cells (with system B [see Materials and methods]). The highest number of fluorescent spots was obtained at the concentration of 5 μg/mL for RNP and 3.3 μg/mL for plasmid DNA (Figure 5C). This experiment suggested that the RNP-based delivery using 1.7~5 μg/mL Cas9 protein yields a higher editing outcome than the plasmid-based delivery in the hydrodynamic injection method. We further confirmed the results of hydrodynamic injection using plasmid (3.3 μg/mL) or RNP (5 μg/mL) by quantifying EGFP-positive cells in liver sections. EGFP+/4′,6-diamidino-2-phenylindole (DAPI) ratios were about 5% for plasmid and about 8% for RNP, showing that the number of fluorescent cells via RNP delivery was 1.6-fold higher than that achieved by plasmid DNA (Figure 5D). We also employed a semi-quantitation approach of counting green spots in the liver images, and the results were comparable (1.9-fold [719 for plasmid (3.3 μg/mL) and 1,332 for RNP (5 μg/mL)]) (Figure 5C).

Lower efficiency of plasmid-based delivery, compared to RNP delivery, could be because the kinetics of genome editing by plasmid is slower, considering that Cas9 expression may take a longer time to reach maximal the Cas9 protein level. To rule out this possibility, we examined the fluorescence in the liver 15 days after hydrodynamic delivery of pP189. The results showed no difference in the number of fluorescent spots between the liver sample collected on days 4 and 15 (282 ± 103 and 245 ± 46 for 4 days and 15 days after the delivery, respectively [with system A]). These data suggested that the RNP system works more efficiently than the vector-based gene expression system for genome editing.

### *In vivo* DNA cleavage efficiencies of gRNA-Cr1 and -Cr6

The number of fluorescent spots obtained by HDR-based gene correction using gRNA-Cr6 and ssODN-2 was lower than the NHEJ-based gene correction using gRNA-Cr1 (Figure 5A). There could be a few reasons for this. First, HDR efficiency is generally lower compared to NHEJ, which is a well-established observation in the field. The second reason is the relative distances of the guides from the site of the ΔEGFP mutation. The cleavage site for gRNA-Cr1 is four bases away, whereas the gRNA-Cr6 is 21 bases away. The further the guide from the target site, the lower the gene editing efficiency in HDR process.^19^ Lastly, it could also be due to lower cleavage efficiency by gRNA-Cr6. To rule out this last possibility, we performed deep sequencing to compare DNA cleavage efficiencies between gRNA-Cr1 and -Cr6 using the DNA sample derived from mouse liver with RNP-based editing. About 11.8% (8,856/75,323) and 6.0% (4,460/74,767) reads contained indel mutations in the samples obtained from gRNA-Cr1 and gRNA-Cr6, respectively (Table S2), indicating that there is only a 2-fold difference in guide-cleaving activity (based on the indel frequencies). The difference of low HDR efficiencies shown in Figure 5A may not be solely due to the difference in the guide-cleavage efficiencies. Taken together, the relative differences in fluorescence restoration between the gRNA-Cr1 and gRNA-Cr6 could be a combined effect of all three reasons. It is noteworthy that very low knock-in efficiency (0.005% [4/74,767]) was detected with the “gRNA-Cr6 and ssODN-2” combination via deep sequencing analysis (Table S2), whereas fluorescence-based detection using microscopy could readily detect HDR outcomes in the liver (Figure 5A).

### Identification and analyses of genome-edited alleles in liver and oviduct

We further investigated the results of genome editing by analyzing the *EGFP* gene sequences in fluorescent-positive (EGFP+) and -negative (EGFP−) hepatocytes. The primary hepatocytes were isolated and subjected to flow cytometry analysis 3 days after hydrodynamic treatment with RNP gRNA-Cr1 (5 μg/mL), which revealed that the percentage of EGFP+ cells was about 4.6% (Table 1; Figure S3). Deep-sequencing analysis of the target region in each EGFP+/− cell revealed that 63.3% alleles in EGFP+ cells had indel mutations, and 26.9% (42.5% of all indels) were frame-restored indels (in-frame indels) (Tables 1 and S3). On the other hand, we found that 7.5% alleles in EGFP− cells had indels, and unexpectedly, 1.1% (15.0% of all indels) were in-frame indels. Based on the proportion of EGFP+ cells (4.6%), the approximate rate of indels in the total hepatocyte was calculated to be 10.1%, and the rate of in-frame sequences was 2.3% (23.4% of all indels) (Tables 1 and S3). In addition, we found that the top three indel sequences accounted for about 70% of the total indels (Table S4). For in-frame indels, the most frequently detected sequence in EGFP− cells was also the most abundant in EGFP+ cells (indel #3 in Table S4 and the same sequence as allele 2 in Figure 3E). Indel #3 sequence was found to fluoresce after the hydrodynamic delivery of its expression construct (Figure S4), suggesting that the genome editing had just occurred in the indel #3-containing EGFP− cells, and the cells were not yet ready to express fluorescence at 3 days post-hydrodynamic injection.

**Table 1:** Deep sequencing analysis of indel mutation rate in liver and oviduct^a^

	EGFP fluorescence	Indel/reads (%)	In-frame/reads (%)	In-frame/indel (%)
Liver (n = 3)	+ (4.6%)	63.3	26.9	42.5
	− (95.4%)	7.5	1.1	15.0
	+/−	10.1	2.3	23.4
Oviduct (n = 2)	+/−	4.4	0.8	16.7

a See also Table S3 for detail.

We also performed deep-sequencing analysis for the regions of the oviduct showing fluorescence. The frequently detected indels in the liver were abundant in the oviduct (Table S4). The in-frame indels accounted for 16.7% of all indels (Tables 1 and S3).

### Use of Cas12a (also known as Cpf1) derived from *Acidaminococcus sp.* (AsCas12a) for detection of HDR

The AsCas12a is an alternative nuclease often used in research. We designed AsCas12a gRNA at the region close to the ΔEGFP mutation (Figure S5A; Table S1) and examined whether the AsCas12a-based approach can restore EGFP fluorescence in #197 mice. *In vivo* electroporation and hydrodynamic delivery experiments resulted in restoration of EGFP expression in oviducts and liver cells via HDR (not via NHEJ), respectively, i.e., only when ssODN-2 was included in the delivery components (Figure S5B), confirming that the ΔEGFP Tg mouse model can also be used as a reporter for evaluation of *in vivo* genome editing by AsCas12a.

## Discussion

In this study, we produced two ΔEGFP Tg mouse lines by creating frameshift mutations in the *EGFP* sequence of our previously developed single-copy EGFP Tg mouse.^17^ One model has a 1-bp deletion (line 197), and the second model has a 13-bp deletion (line 201). Both ΔEGFP models lost EGFP expression and fluorescence in the entire body, and the functional EGFP was restored in the fertilized eggs or organs (such as oviducts and liver) using the CRISPR-Cas9 genome editing tool. We demonstrated that the level of EGFP expression in the liver is dependent on the types of gene-editing components delivered, with RNP exhibiting better efficiency than plasmid vector-based systems. The study also showed that the ΔEGFP mice (line #197) would be useful to evaluate not only HDR-based knock-in but also a NHEJ-based repair system. Two different gRNAs were evaluated, one suitable for NHEJ (gRNA Cr1) and the other for HDR (gRNA Cr6) using SpCas9 nuclease. In addition, the ΔEGFP model (line 197) is also suitable for evaluation of AsCas12a nuclease-based genome editing.

We observed that ssODN-1 for HDR-based repair was not a strict requirement for eliciting green fluorescence in ΔEGFP mice (line #197) when gRNA-Cr1 was used, suggesting the involvement of a NHEJ-based repair mechanism. We confirmed that EGFP fluorescence was recovered when the indel mutation restored the reading frame of the *EGFP* gene. On the other hand, the indel mutation allele did not restore EGFP fluorescence when the reading frame was not corrected, suggesting that some cells that do not emit EGFP may still be genome edited (see below for more about this).

We determined near-optimal concentration of CRISPR components (for both plasmid and RNP) to elicit highest genome editing via the hydrodynamics delivery approach. To our knowledge, this is the first report demonstrating that the hydrodynamics delivery of RNP of Cas9 and gRNA into the liver is more effective than the plasmid vector expressing Cas9 and sgRNA to achieve *in vivo* gene editing. It is possible that the formation of functional RNP in vector-transfected cells is less efficient compared to direct injection of the preassembled RNPs. Another possibility is that hydrodynamic delivery of RNP is more efficient than the plasmid DNA. It is noteworthy that, from our observation of various liver sections, we did not detect any preference of higher/lower genome editing efficiency in different regions of liver, irrespective of the delivery methods used (RNP or plasmid DNA based). One of the major advantages of the RNP-based system is that it is rapid and transient; thus, the chances of off-target effects can be minimal compared to plasmid vectors that can remain in the cells or tissues for longer durations.^20^^,^^21^

To understand the genome editing efficiencies, we quantified and evaluated the percentage of EGFP+ cells in the liver by three different methods: (1) counting the number of EGFP+ spots in images of dissected liver lobes, (2) counting EGFP+ cells in frozen sections, and (3) flow cytometry analysis. Of these, (2) and (3) allowed for absolute quantification, whereas (1) is considered to be an approximate quantification. When plasmid (3.3 μg/mL) and RNP (5 μg/mL) were compared, the ratio was 1:1.9 (plasmid = 719; RNP = 1,332) in method 1 and 1:1.6 (plasmid = 5%; RNP = 8%) in method 2, confirming that comparable results were obtained with both of the methods. Although the absolute quantification method 2 is considered to be more accurate, method 1 may be advantageous when a large number of relative comparisons need to be made between different concentrations or formats. We would like to note that differences in ImageJ settings (in method 2) or some bias in isolation of hepatocytes and setting of sorting conditions (in method 3) can contribute to some variations in readings. For example, when the percentage of EGFP+ cells in RNP (5 μg/mL)-transfected livers was examined with methods 2 and 3, the percentage of EGFP+ cells in (2) was about 8%, and that in (3) was about 4.6%.

After 3 days of hydrodynamic delivery of gRNA-Cr1 RNP (5 μg/mL), 63.3% of the alleles in the EGFP+ cells contained indels; 42.5% of those indels (26.9% of all the alleles) were frame-restored edits, and the remaining (57.5%) were out-of-frame edits. With the consideration that about 80% to 90% of hepatocytes in adult mice are polyploid (mainly tetraploid),^22^ more than 1/4 (26.9%) EGFP+ cell-contained in-frame edits seems to be a notable efficiency. These results suggested that all EGFP+ cells have, on average, at least one in-frame indel, together with one or two frameshift indel alleles. On the other hand, 7.5% of the analyzed sequences contained indel mutations in EGFP− cells. With the assumption that the majority of hepatocytes are tetraploid and that two to three out of four alleles in genome-edited EGFP− cells have indel mutations, as in EGFP+ cells, about 12.5% of EGFP− cells are considered to have indels. Since 4.6% of hepatocytes were EGFP+ cells, it can be assumed that genome editing occurred in about 17.1% (12.5% + 4.6%) of the total hepatocytes in the RNP-based hydrodynamic delivery. The hypothetical genome editing outcome of the liver in 3 days after hydrodynamic treatment is illustrated in Figure S6.

The concept of our reporter mouse models is similar to a work published previously that used a LacZ reporter (unlike the EGFP reporter used in our study).^23^ Nickerson and Colledge^23^ created a delta-lacZ (Δ1 nt) knock-in mouse and examined whether this mouse model could be applicable for evaluating gene delivery efficiency, using lacZ staining for detection. The attempt was not successful because they were unable to detect lacZ signals in various tissues, even when different delivery methods were tested. The authors concluded that the repair and/or delivery efficiency could be too low to detect the lacZ signal. Their system would work as expected with the current CRISPR system. However, the EGFP-based reporter system generated in this study offers advantages over lacZ reporters, because tissue fixation and chemical staining steps are not required for visualization. Furthermore, both NHEJ and HDR are detected in our ΔEGFP reporter mice. Two reports utilized Cre reporter mouse strains, such as Ai9 and Ai14, as the *in vivo* CRISPR activity reporter, by administrating two gRNAs targeting sequences close to each *loxP* site to make excision of the floxed STOP cassette containing the polyadenylation sequence.^24^^,^^25^ This system is not based on the concept of reading-frame shifting, and therefore, it can only detect NHEJ events. It is likely that not all NHEJ outcomes can be detected, even with this system, because occurrences of only single *loxP* editing or inversions between the two *loxP*s events can go undetected. To our knowledge, this system has not been applied for detecting HDR-based knock-in and for AsCas12a-based genome editing, so far. Furthermore, ΔEGFP mice could be used to evaluate other genome-editing enzymes, depending on the protospacer adjacent motif (PAM) sequence. For example, gRNA targets for the recently reported CasPhi-2 (PAM sequence “TBN”)^26^ can be designed in the same region as AsCas12a (PAM sequence “TTTN”). Therefore, it is expected that ΔEGFP mice can also be used for the evaluation of CasPhi-2.

The CRISPR genome editing system has great potential as an effective tool for gene therapy.^15^ The remaining challenges in the field are as follows: (1) development of new strategies for efficient delivery of CRISPR components to target cells and (2) development of new editing components eliciting high genomic editing efficiency with low off-target effects. The reporter mouse models that we developed in this study serve as valuable tools for developing and evaluating gene editing technologies and for advancing gene therapy research.

## Materials and methods

### Mice

The EGFP Tg mouse line (B6.Cg-Gt(ROSA)26Sor<tm2.1(CAG-EGFP)Maoh>), containing a single copy of the *EGFP* expression cassette inserted into the *Rosa26* locus,^17^ was used for generating the ΔEGFP Tg mouse lines #197 and #201, and the parental strain was also used as a positive control for FACS analysis. The line #197 (B6.129P2-Gt(ROSA)26Sor<em1(CAG-EGFP<∗1>)Maoh>) is now available at RIKEN BioResource Research Center (RBRC10181). C57BL/6J female mice (8 weeks old), used as embryo donors, and MCH(ICR) female mice (10 to 20 weeks old), used as the foster mother, were purchased from CLEA Japan (Tokyo, Japan). The mice were kept on a 12-h light/12-h dark schedule (lights on from 07:00 to 19:00) and allowed food and water *ad libitum*. All of the animal experiments were performed in accordance with institutional guidelines and were approved by the Institutional Animal Care and Use Committee (permit nos. 154014, 165009, 171003, 182032, 193010, and 204019 at Tokai University; AUP:2639 [A2017 06-005-Y1-A0] at the University of Georgia). All efforts were made to minimize the number of animals used and animal suffering.

### Genome editing components and vectors used in this study

We designed gRNA sequences using CHOPCHOP (Table S1).^27^ Three sets of oligo pairs (PP170 and PP171 for gRNA-Cr1, PP176 and PP177 for gRNA-Cr4, and PP244 and PP245 for gRNA-Cr6; Table S5) were commercially synthesized and cloned into the BbsI site of the pX330 vector (Addgene; plasmid no. 42230),^28^ which confers simultaneous expression of both Cas9 and sgRNA. The plasmids were named pP189 and pP217 for sgRNA-Cr1 and sgRNA-Cr6 expression, respectively. The plasmids were purified using an Endo-Free Plasmid Purification Kit (QIAGEN) prior to *in vivo* gene delivery experiment. For generating the pP216 construct, we first amplified mCherry cDNA with a primer set (M1070 and M1071; Table S5) using pAOB plasmid as a template,^17^ and the amplified fragment was inserted into AgeI/EcoRl sites of pGFPuv expression plasmid from Clontech.

The gRNA, sgRNA-EGFP, which had been successfully used for inducing mutations in *EGFP* cDNA *in vivo*,^29^ was used for disrupting the *EGFP* coding region of the EGFP Tg mice. Other sgRNA (Cr1 and Cr4) used for repairing the disrupted *EGFP* gene (ΔEGFP) were synthesized from PCR products using the plasmids pP189 and pP192 as a template and primer sets M939-PP184 or M939-PP200, respectively. All sgRNAs and *Cas9* mRNA were synthesized as described in our previous work.^29^

crRNAs (Cr1, Cr6, and Cr7); tracrRNA; gRNA for AsCas12a, ssODN-1, and ssODN-2 (used for editing of ΔEGFP); and Cas9 protein (SpCas9) were purchased from Integrated DNA Technologies (IDT), and crRNAs and tracrRNA were annealed to obtain functional gRNAs and mixed with Cas9 protein to obtain ctRNP complexes, as described previously.^21^^,^^30^ AsCas12a Ultra was a gift from IDT.

### Microinjection

To obtain ΔEGFP Tg mice, a solution containing *Cas9* mRNA (20 ng/μL) and sgRNA-EGFP (10 ng/μL) was injected into pronucleus and cytoplasm of fertilized eggs, which were obtained through *in vitro* fertilization (IVF) between C57BL/6J-derived oocytes and homozygous EGFP Tg-derived spermatozoa. The injected eggs were then transferred to oviducts of pseudopregnant MCH(ICR) female mice.

To correct the frameshift mutations in the ΔEGFP Tg lines, pronuclear and cytoplasmic injections of a solution containing Cas9 mRNA (5 ng/μL), sgRNA-Cr1 or -Cr4 (5 ng/μL), and ssODN-1 (10 ng/μL) or Cas9 protein (0.305 μM), crRNA/tracrRNA format of gRNA-Cr6 or -Cr7 (0.61 μM), and ssODN-2 (10 ng/μL) were performed into fertilized eggs obtained through IVF between C57BL/6J-derived oocytes and homozygous ΔEGFP Tg (#197 or #201)-derived spermatozoa. After injection, eggs were allowed to develop into blastocysts *in vitro* in a KSOM medium.

### Intra-oviductal gene delivery

Gene delivery into oviductal epithelium was performed using the procedures described in our improved genome editing via oviductal nucleic acids delivery (i-GONAD) method.^31^ Briefly, homozygous ΔEGFP Tg female mice were anesthetized by intraperitoneal (i.p.) or subcutaneous (s.c.) injection of the combination of three anesthetics (medetomidine hydrochloride, midazolam, and butorphanol tartrate). The ovary and the oviducts were exposed, and 1.5 μL of a solution containing a mixture of Cas9 protein (1 μg/μL) and crRNA/tracrRNA (30 μM each) (with or without ssDNA-1 or -2 [2 μg/μL]) was injected into the oviductal lumen. For the experiment using AsCas12a, 1.5 μL of a solution containing a mixture of AsCas12a (1 μg/μL), gRNA-Cas12a (30 μM), and ssDNA-2 (2 μg/μL) was injected into the oviductal lumen. Immediately after injection, the entire oviduct was subjected to *in vivo* electroporation using an electroporator (CUY21Editll; BEX) with tweezer-type electrodes (LF650P3; BEX). The electroporation conditions were as follows: square (mA), (+/−), Pd V: 80 V, Pd A: 150 mA, Pd on: 5.00 ms, Pd off: 50 ms, Pd N: 3, decay: 10%, DecayType: Log.^31^ After electroporation, the tissues were returned to the original position, and the incision was closed by surgical clips. The anesthetized mice were recovered by i.p. or s.c. injection of atipamezole, an antagonism of medetomidine, and then warmed using an electric plate warmer. The mice were housed for about 3 days, 1 week (for Cas9), or 13 days (for Cas12a) and were used for analysis.

### Hydrodynamic delivery

Hydrodynamic delivery was performed following the procedure as previously reported.^32^ In brief, hemizygous (for experiments in Figures 5A and 5B, and Table S2) or homozygous (for experiments in Figures 5C, 5D, S3 and S5, and Tables 1, S3 and S4) ΔEGFP Tg mice placed in the mouse holder (Braintree Scientific) were injected with a solution (one-tenth of the mouse weight in volume; for example, 2.3 mL/23 g of a mouse) containing pP189 or pP217 plasmid (0.2~30 μg/mL) or Cas9 protein (0.5~5 μg/mL) and tracrRNA/crRNA (15~150 nM) with or without ssODN (10 μg/mL) by a syringe (3 mL Luer-Lok type; Becton Dickinson [BD]) fitted with a 27-gauge needle (BD). For the experiment using AsCas12a, a solution containing a mixture of AsCas12a (1.7 μg/mL), gRNA-Cas12a (50 nM), and ssODN-2 (3.3 μg/mL) was injected. Injections were performed at a constant injection speed via tail vein and completed at around 5 s. 3 to 16 days later, EGFP fluorescence in the livers was analyzed. Hydrodynamic delivery of pP233 plasmid (3.3 μg/mL), containing “CAG promoter-EGFP (indel #3)-pA” cassette, was also performed. 3 days later, EGFP fluorescence in the livers was analyzed.

### Fluorescence observation

Expression of EGFP in the fertilized eggs was observed under an Olympus IX70 inverted fluorescence microscope (Olympus) with filter sets (U-MNIBA). The EGFP fluorescence in various tissues, including oviducts, was observed under fluorescence stereomicroscope, with filter for GFP (Olympus SZX7 with SZX-MGFPA), and fluorescent images were acquired using EOS Kiss X5. The area of fluorescent signal in the image was quantified using ImageJ software and expressed as percentages. Fluorescence in adult live mice was observed under a long-wave UV light and photographed by digital camera (EOS Kiss X5; Canon). To prepare spleen cells for fluorescence analysis, the contaminating red blood cells were cleared by osmotic lysis solution (RBC Lysis Buffer 00-4300-54; eBioscience), and the unlysed spleen cells were resuspended in complete medium. After filtration using a 35-μm mesh filter (Falcon 352235; BD), the cells were subjected to flow cytometry analysis by LSRFortessa (BD). The EGFP fluorescence in liver (following hydrodynamic delivery) was observed under fluorescence stereomicroscope with a filter for GFP, and fluorescent images were acquired (more than five images [each from a different region of the liver]/mouse). Two systems were used for image acquisition: system A: SteREO Discovery.V8 stereomicroscope and microscope-bundled software (ZEN; Zeiss) in the University of Georgia, and system B: Olympus SZX7 and EOS Kiss X5 in Tokai University. The number of fluorescent spots in the images was counted using ImageJ software and then manually confirmed for each image.

### Genotyping analyses

A piece of mouse ear was used for isolating genomic DNA by immersing it in 40 μL of All-In-One Mouse Tail Lysis Buffer (Kurabo) and incubating it at 55°C for 3 h. After the inactivation of proteinase at 85°C for 30 min, the isolated genomic DNA (1 μL) was used for the PCR assay in a total volume of 10 μL containing 1 × GC buffer, 0.5 μM primers (M212 and M026; Table S5), 200 μM deoxyribonucleotide triphosphate (dNTP), and 0.125 units of r-Taq (TaKaRa). Reactions were performed in a thermal cycler (Mastercycler nexus; Eppendorf) with the following conditions: 95°C for 5 min, followed by 95°C for 45 s, 58°C for 30 s, and 72°C for 1 min for 35 cycles and 72°C for 5 min. 5 μL of each PCR product was separated on a 1% agarose gel and then stained with ethidium bromide (EtBr) for DNA visualization. After treatment with ExoSAP, PCR products were used as in direct sequencing reactions.

For genotyping of blastocysts, each embryo was transferred from the culture dish to a MicroWell MiniTray (Thermo Scientific) using an egg-handling pipette. After removing the remaining culture medium, each well was filled with 10 μL of All-In-One Mouse Tail Lysis Buffer, and the solution from each well was transferred to 0.2 mL PCR tubes. The tubes were incubated at 55°C for 3 h and then treated at 85°C for 10 min for inactivation of proteinase. The crude lysate (1 μL) was used for PCR in a total volume of 10 μL solution containing 1 × LA buffer and 2.5 mM MgCl_2_ (or 1 × GC buffer), 1 μM primer set (M212 and M495; Table S5), 375 μM dNTP, and 0.25 units of La-Taq (TaKaRa). Nested PCR was then performed in a total volume of 10 μL containing 1 × GC buffer, 0.5 μM primers (M389 and M026; Table S5), 200 μM dNTP, 0.5 μL of 1^st^ PCR product, and 0.125 units of r-Taq (TaKaRa). The PCR conditions were the same as those used for analyzing ear DNA. 5 μL of each PCR product was separated on a 1% agarose gel and then stained with EtBr for DNA visualization. Direct sequencing of the PCR products with primer M026 was performed after ExoSAP treatment.

### Sequencing of the modified ΔEGFP gene capable of showing green fluorescence

Oviducts showing EGFP-derived fluorescence after intra-oviductal delivery of RNP (for gRNA-Cr1) into ΔEGFP Tg mice were dissected and subjected to genomic DNA isolation by immersing them in All-In-One Mouse Tail Lysis Buffer and then incubated at 55°C for 3 h and at 85°C for 10 min. The genomic DNA isolated was then subjected to PCR using the M1070 and M1071 primer set (Table S5), prior to sub-cloning into the AgeI and EcoRI sites of the pP216 vector, a vector conferring expression of a cloned fragment in *E. coli* (DH5α competent cells; TaKaRa). Fluorescence in generating *E. coli* colonies was directly inspected under a fluorescence stereomicroscope, with filter for green and red fluorescence, and the number of colonies showing red, green, and no fluorescence was recorded. Next, a few green fluorescent colonies were picked and subjected to “colony PCR” using a “M328 and M880” primer set (Table S5). Direct sequencing of the PCR products was then performed.

### Mouse primary hepatocyte isolation

Hepatocytes were isolated from mice by two-step collagenase perfusion, according to Hu et al.^33^ Briefly after placing the catheter (Terumo; SR-FS2225) into the portal vein, the inferior vena cava was cut, and the liver was perfused at 6–7 mL/min, with 50 mL pre-warmed perfusion medium. Perfusion was then performed with 50 mL pre-warmed collagenase solution buffer, including collagenase and Ca^2+^ at 6–7 mL/min. After dissociation, cells were filtered with a cell strainer (Falcon; 3S2340). Hepatocytes were further separated and purified by centrifugation at low speed (50 g, 2 min), and Percoll gradient centrifugation was performed. Cells were resuspended with 1 mL medium for FACS analysis.

### FACS analysis and cell sorting

Primary hepatocyte was analyzed and sorted on a fluorescence-activated cell sorter, FACSAria (BD Biosciences), by measuring GFP fluorescence. Percentage of GFP+/− cells was determined by analyzing at least 10^6^ cells per sample.

### Tissue sections

Trimmings of liver tissue and oviduct parts showing EGFP expression were performed. Frozen sections (5 μm size) were prepared as previously described,^34^ and the sections were imaged for fluorescence after staining with DAPI.

### Next-generation sequencing (NGS)

Genomic DNAs were isolated from liver tissues using the DNeasy Blood and Tissue Kit (QIAGEN). The PCR was performed with a primer set (M026 and M212; Table S5). The PCR reaction mixture contained 200 ng of genomic DNA, 1 × KOD buffer, 0.4 μM primers, 200 μM dNTP, 150 μM MgSO_4,_ and 0.001 units of KOD (Toyobo) in a final volume of 50 μL. The PCR conditions were as follows: initial denaturation at 95°C for 1 min, 27 thermal cycles of denaturation at 95°C for 15 s, and extension at 68°C for 30 s, followed by final incubation at 68°C for 7 min. After purification of PCR products with NucleoSpin Gel and PCR Clean-up (TaKaRa), these (100 ng) were end repaired, dA tailed, and ligated with a SeqCap Adopter (Nippon gene) for Illumina sequencing using KAPA HTP/LTP Library Preparation Kits (Roche), according to the manufacturer’s instructions. The second PCR reaction mixture contained 20 μL of adaptor-ligated DNA products, 5 μL of Library Amplification Primer Mix, and 25 μL of KAPA HiFi HotStart ReadyMix in a final volume of 50 μL. The second PCR conditions were as follows: initial denaturation at 98°C for 45 s, 4 thermal cycles of denaturation at 98°C for 15 s, primer annealing and extension at 60°C for 30 s, and 72°C for 30 s, followed by final incubation at 72°C for 1 min. The resulting PCR products were purified with AMPure XP. Each adaptor-conjugated amplicon was quantified by Qubit dsDNA HS Assay Kit (Thermo Fisher Scientific), and the sizes and purities were verified by Bioanalyzer. Each sample was normalized, pooled at an equimolar amount, and mixed with 25% PhiX Control V3 (Illumina). These libraries, consisting of nine samples each, were sequenced on the Illumina MiSeq with MiSeq Reagent Kit version (v.)2 (300 cycles; Illumina).

### NGS data analysis

Amplicon sequencing reactions were analyzed as follows: demultiplexed FASTQ files were aligned to predicted sequence files for unedited (wild-type [WT] sequence) and edited amplicons using the Cas-Analyzer online tool (http://www.rgenome.net/cas-analyzer/#!), using a parameter (comparison range [R]: 50; minimum frequency [n]: 1; WT marker [r]: 5, or R: 30; n: 0; r: 7).^35^ Raw data were extracted and replotted as required.

## Supplemental information

Document S1. Tables S1–S5 and Figures S1–S6

Document S2. Article plus supplemental information
