# Supplementary material for: Novel reporter mouse models useful for evaluating in vivo gene editing and for optimization of methods of delivering genome editing tools
Source: Mol Ther Nucleic Acids. 2021 Mar 5;24:325–36. doi: 10.1016/j.omtn.2021.03.003 (PMC8020343; doi:10.1016/j.omtn.2021.03.003)
Supplement: Document S1. Tables S1–S5 and Figures S1–S6 [file mmc1.pdf]

**OMTN, Volume *24***

## Supplemental information

# Novel reporter mouse models useful for evaluating *in vivo* gene editing and for optimization of methods of delivering genome editing tools

**Hiromi Miura, Jurai Imafuku, Aki Kurosaki, Masahiro Sato, Yongjie Ma, Guisheng Zhang, Akiko Mizutani, Kenya Kamimura, Channabasavaiah B. Gurumurthy, Dexi Liu, and Masato Ohtsuka**

| **Table S1.** CRISPR target sequences | |
|---|---|
| **Name of gRNA** | **Sequence (5'-3')** |
| sgRNA-eGFP | GACCAGGATGGGCACCACCC [CGG (PAM)] |
| gRNA-Cr1 | GCGAGGAGCTGTTCACCGGG [GGG (PAM)] |
| gRNA-Cr4 | TGTGCCCATCCTGGTCGAGC [TGG (PAM)] |
| gRNA-Cr6 | CCCATCCTGGTCGAGCTGGA [CGG (PAM)] |
| gRNA-Cr7 | GAGCTGGACGGCGACGTAAA [CGG (PAM)] |
| gRNA-Cas12a | [TTTA (PAM)] CGTCGCCGTCCAGCTCGACCAGGA |

| **Table S2.** Deep sequencing analysis of the number of induced mutations by two different gRNAs | | | |
|---|---|---|---|
| **CRISPR reagents used (N*)** | **Total no. of reads analyzed** | **No. of indel** | **No. of knock-in** |
| gRNA-Cr1 (3) | 75323 | 8856 | 0 |
| gRNA-Cr6 + ssODN-2 (4) | 74767 | 4460 | 4 |
| *Number of mice used for analysis | | | |

**Table S3.** Deep sequencing analysis of indel mutation rate in liver and oviduct

| | Exp. | eGFP fluorescence | Reads | Indel | Indel /Reads | In-frame | In-frame /Reads | In-frame /Indel |
|---|---|---|---|---|---|---|---|---|
| **Liver** | 1 | + (5.1%) | 18258 | 11037 | 60.5% | 4751 | 26.0% | 43.0% |
| | | - (94.9%) | 25409 | 1316 | 5.2% | 244 | 1.0% | 18.5% |
| | | +/- | | | 8.0% | | 2.3% | 28.8% |
| | 2 | + (4.7%) | 19732 | 12924 | 65.5% | 5420 | 27.5% | 41.9% |
| | | - (95.3%) | 27356 | 2868 | 10.5% | 324 | 1.2% | 11.3% |
| | | +/- | | | 13.1% | | 2.4% | 18.3% |
| | 3 | + (3.9%) | 18733 | 11968 | 63.9% | 5100 | 27.2% | 42.6% |
| | | - (96.1%) | 20657 | 1425 | 6.9% | 217 | 1.1% | 15.2% |
| | | +/- | | | 9.1% | | 2.1% | 23.1% |
| | **Average** | **+ (4.6%)** | | | **63.3%** | | **26.9%** | **42.5%** |
| | | **- (95.4%)** | | | **7.5%** | | **1.1%** | **15.0%** |
| | | **+/-** | | | **10.1%** | | **2.3%** | **23.4%** |
| **Oviduct** | 1 | | 3285 | 155 | 4.7% | 38 | 1.2% | 24.5% |
| | 2 | | 4380 | 180 | 4.1% | 16 | 0.4% | 8.9% |
| | **Average** | | | | **4.4%** | | **0.8%** | **16.7%** |

| **Table S4.** Frequently detected indel alleles in deep sequencing analyses | | | | |
|---|---|---|---|---|
| Indels | Sequences | Editing outcome | Liver* | Oviduct* |
| #1 | ATGGTGAGCAAGGGCGAGGAGCTGTTCA-CGGGGGGTGCCCATCCTGGTCGAGCTGGACG | frameshift | 45.4% | 36.0% |
| #2 | ATGGTGAGCAAGGGCGAGGAGCTGTTCACC-GGGGGTGCCCATCCTGGTCGAGCTGGACG | frameshift | 13.3% | 12.1% |
| #3 | ATGGTGAGCAAGGGCGAGGAGCTGTTCACCCGGGGGGTGCCCATCCTGGTCGAGCTGGACG | In-frame | 9.6% | 6.2% |
| * The frequencies among the indel mutations are shown | | | | |

| **Table S5.** Sequences of the oligonucleotides used in this study | |
|---|---|
| **Name of oligos** | **Sequences (5'-3')** |
| ssODN-1 | GCAACGTGCTGGTTATTGTGCTGTCTCATCATTTTGGCAAAGAATTGGGATCCACCGGTCGCCACCATGGTGAGCAAGGGCGAGGAGCTGTTCACCGGGGTGGTGCCCATCCTGGTCGAGCTGGACGGCGACGTAAACGGCCACAAGTTCAGCGTGTCCGGCGAGGGCGAGGGCGATGCCACCTACGGCAAGCTGACCCT |
| ssODN-2 | CATTTTGGCAAAGAATTGGGATCCACCGGTCGCCACCATGGTGAGCAAGGGCGAGGAGCTGTTCACCGGGGTGGTGCCCATCCTGGTCGAGCTCGACGGCGACGTAAACGGCCACAAGTTCAGCGTGTCCGGCGAGGGCGAGGGCGATGCCACCTACGGCAAGCT |
| PP170 | caccGCGAGGAGCTGTTCACCGGG |
| PP171 | aaacCCCGGTGAACAGCTCCTCGC |
| PP176 | caccTGTGCCCATCCTGGTCGAGC |
| PP177 | aaacGCTCGACCAGGATGGGCACA |
| PP244 | caccCCCATCCTGGTCGAGCTGGA |
| PP245 | aaacTCCAGCTCGACCAGGATGGG |
| M1070 | GGTACCGGTAGAAAAAATGGTGAGCAAGGGCGAG |
| M1071 | TTGGAATTCATTACTTGTACAGCTCGTCCAT |
| M939 | AAAAAAAGCACCGACTCGG |
| PP184 | TAATACGACTCACTATAGGGCGAGGAGCTGTTCACCG |
| PP200 | TAATACGACTCACTATAGGgTGTGCCCATCCTGGTCGA |
| M212 | CTCCTGGGCAACGTGCTGGT |
| M026 | GGTGGTGCAGATGAACTTCAG |
| M389 | TCGCCACCatggtgagcaagggcgag |
| M328 | cggataacaatttcacacag |
| M880 | gaaaagtgccacctgacgtc |
| M495 | AAGAAGATGGTGCGCTCCTG |

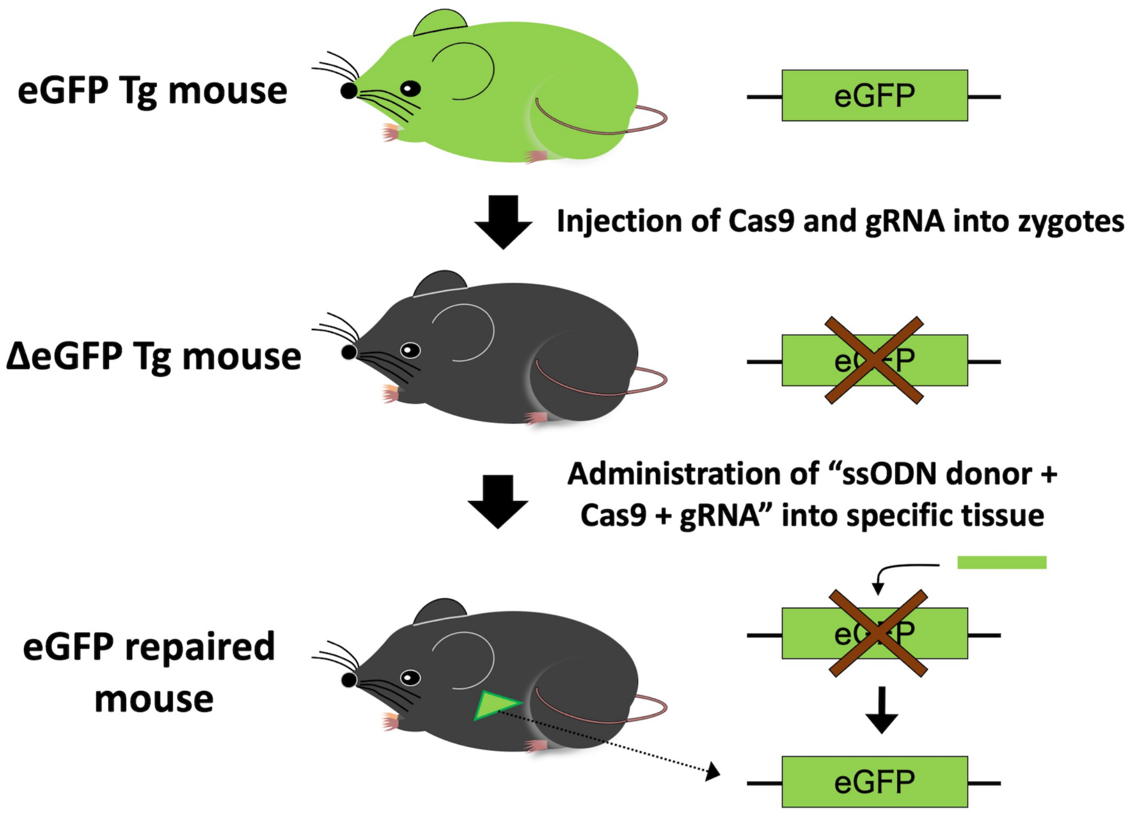


**Figure S1. Schematic of development and use reporter mouse models useful for evaluation of *in vivo* genome editing.** Tg mouse lines containing a defective *eGFP* gene (ΔeGFP Tg mouse) was created by introducing frame-shift mutations into the *eGFP* coding sequence of Tg mouse expressing eGFP ubiquitously [17]. The mouse shown in the top panel represents the parent strain containing a single copy *eGFP* gene that expresses ubiquitously in all tissues. The mouse shown in the middle panel contains a frame-shift mutation (ΔeGFP) and lacks fluorescence. The bottom panel mouse is an example showing restoration of eGFP fluorescence in its liver tissue by delivering CRISPR/Cas9 components (Cas9 and gRNA) and a repair DNA template (ssODN donor) to correct the ΔeGFP mutation.

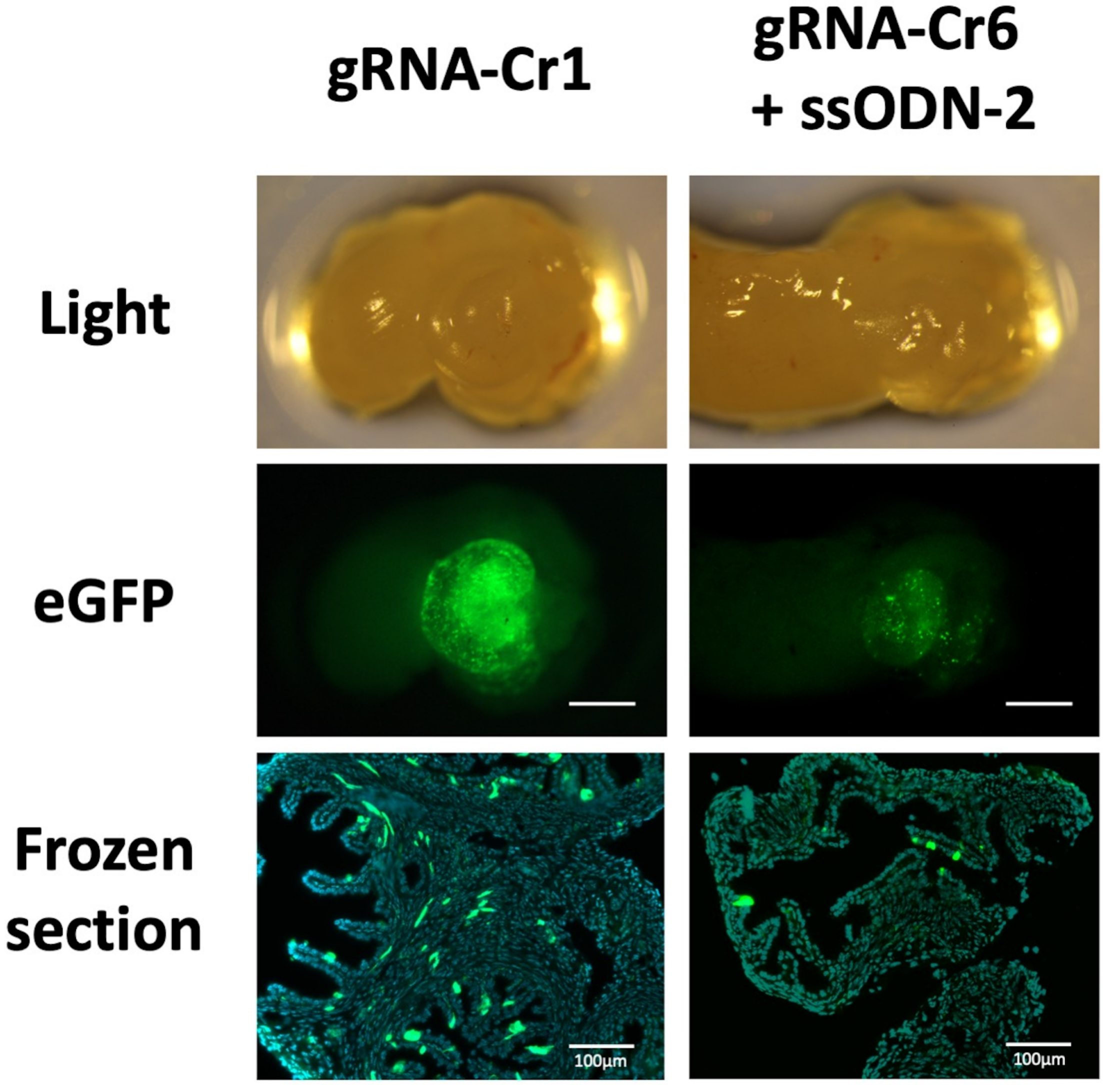


**Figure S2. Restoration of eGFP fluorescence in the oviduct.** Detection of eGFP fluorescence in the oviduct after intra-oviductal injection of CRISPR/Cas9 components (RNP) and subsequent *in vivo* electroporation. The nuclei were stained with DAPI (blue) in frozen section samples. Bar = 1 mm (eGFP) and 100µm (Frozen section).

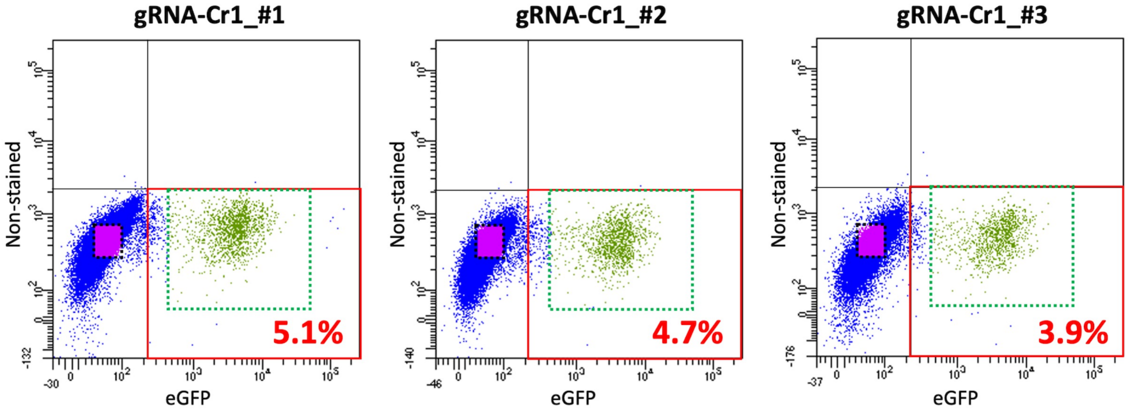


**Figure S3. Flow cytometry analysis of eGFP fluorescence in primary hepatocyte.** Dot plots show the fluorescence intensity of eGFP on a log scale (x-axis). In each plot, the percentage of cells exhibiting eGFP expression in gate Q4 (red rectangle) are shown. The eGFP+ cells in the green-squared region, and eGFP- cells in the black-squared region, were sorted for deep sequencing.

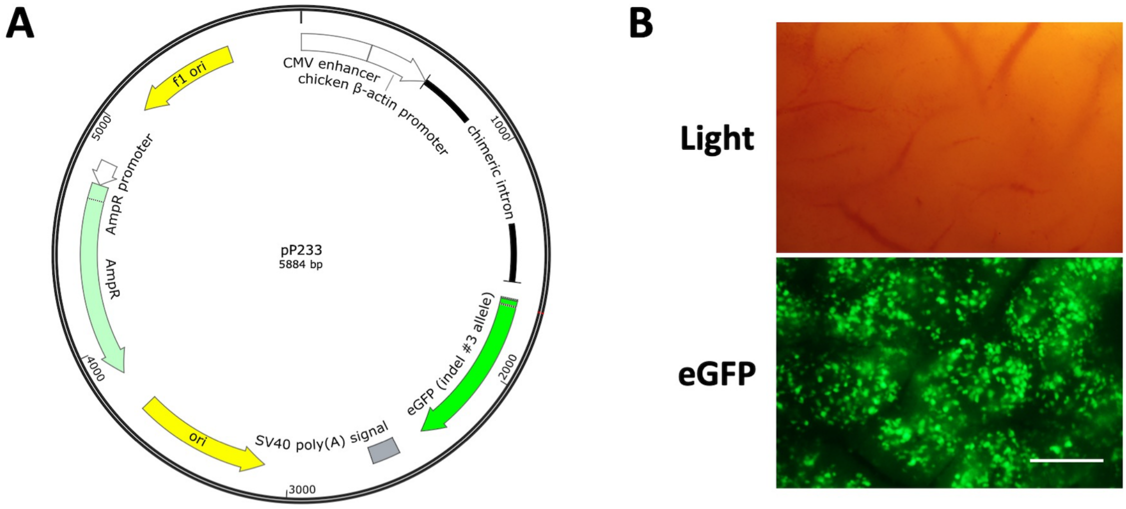


**Figure S4. Fluorescence capability of in-frame allele (Indel #3).** (A) Vector map of pP233, containing eGFP (Indel #3 allele) expression cassette. (B) Detection of eGFP fluorescence in the liver three days after hydrodynamic injection of pP233. Bar = 500 µm.

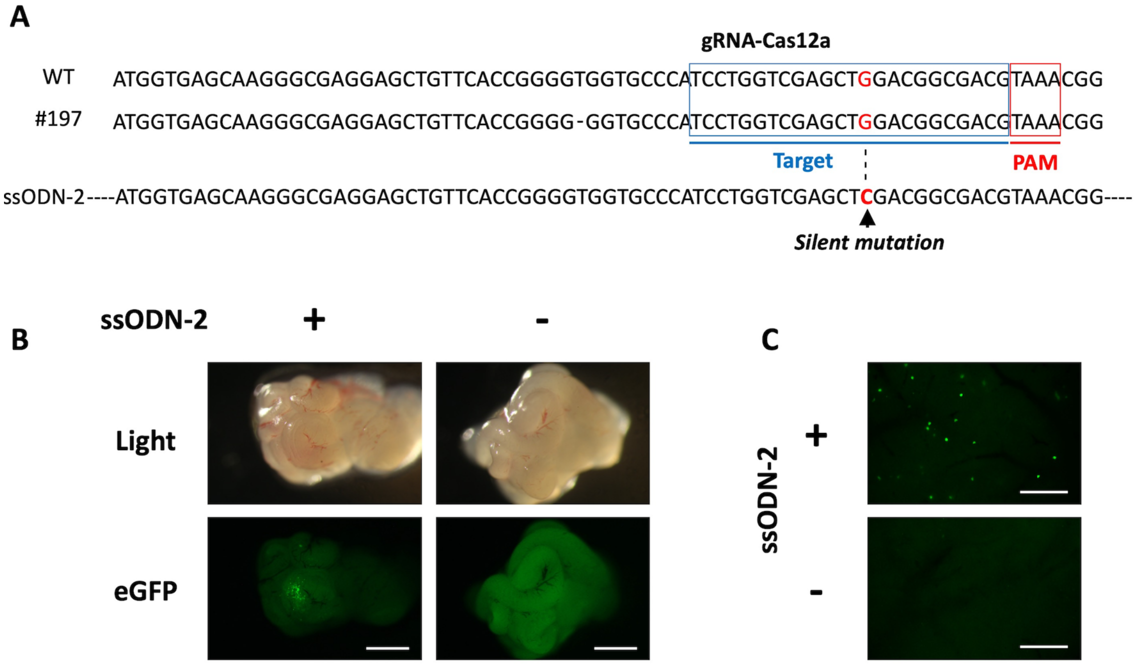


**Figure S5.** ***in vivo*** **restoration of eGFP fluorescence by AsCas12a-based genome editing.** (A) Schematic of the strategy showing correction of ΔeGFP mutation using ssODN-2 after cleavage with AsCas12a. The gRNA target sequences for gRNA-Cas12a and its PAM sequences are indicated by blue and red rectangles, respectively. (B) Detection of eGFP fluorescence in the oviduct after intra-oviductal injection of CRISPR components and subsequent *in vivo* electroporation. Transfection with RNP (containing AsCas12a protein and gRNA-Cas12a) + ssODN-2 was designated as "+", and that with RNP alone as "-". The #197 line was used. Bar = 1 mm. (C) Detection of eGFP fluorescence in the liver after hydrodynamic injection of AsCas12a components. Bar = 500 μm.

A

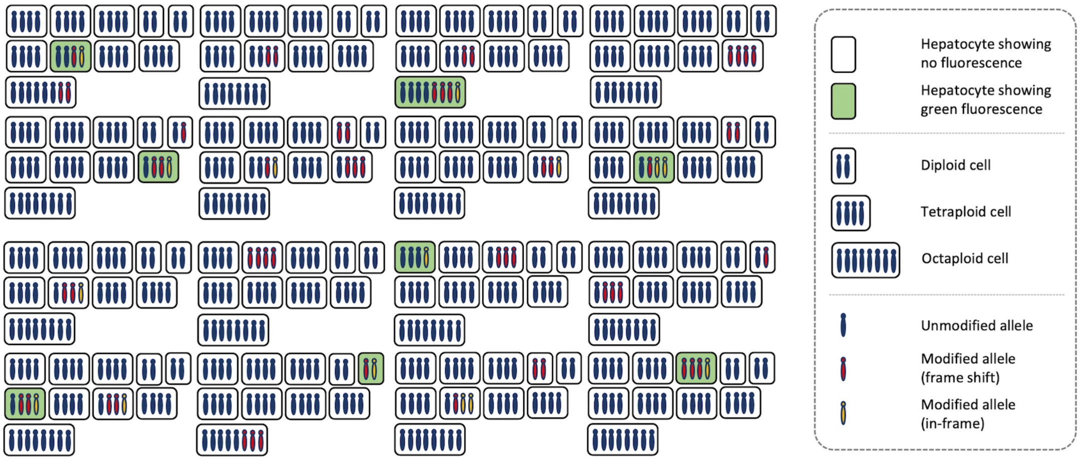


B

| | eGFP fluorescence (% and No. cells) | Indel/Reads | In-frame/Reads | In-frame/Indel |
|---|---|---|---|---|
| Liver | + (5.0% [8/160]) | 64.7% [22/34] | 26.5% [9/34] | 40.9% [9/22] |
| | - (95.0% [152/160]) | 7.9% [48/606] | 1.0% [6/606] | 12.5% [6/48] |
| | +/- [Total 160] | 10.9% [70/640] | 2.3% [15/640] | 21.4% [15/70] |

**Figure S6. Schematic of hypothetical liver three days after hydrodynamic delivery of CRISPR RNP. (**A) Hypothetical outcome of hydrodynamic delivery of RNP (Cas9 protein + gRNA-Cr1). A total of 160 cells consisting of 20% diploid cells, 70% tetraploid cells, and 10% octaploid cells were depicted, based on the Figure 1 of Wang et al., Cell Death Dis (2017). (B) Numbers and percentages of cells, and numbers of indel alleles, are listed based on the hypothetical liver shown in (A). Each % value is close to the value obtained in the actual experiments (see **Table 1**).
